# Amphetamine-related psychosis: what register data reveal?

**DOI:** 10.1192/j.eurpsy.2026.10381

**Published:** 2026-07-31

**Authors:** S. Niemelä, H. Taipale

**Affiliations:** 1 Department of Psychiatry, University of Turku; 2 Mental Health and Addiction Services, Wellbeing Services County of South-West Finland, Turku, Finland; 3 Department of Clinical Neuroscience, Karolinska Institute, Stockholm, Sweden; 4 Department of Forensic Science, Niuvanniemi Hospital, Kuopio, Finland

## Abstract

**Abstract:**

Amphetamine is a common illicit drug in Europe. Heavy use can cause amphetamine-induced psychosis, often mimicking acute paranoid schizophrenia. Differentiating between substance-induced psychosis and primary psychotic disorders with amphetamine use is clinically challenging. Despite frequent presentation in emergency psychiatric care, little is known about the long-term prognosis of amphetamine-related psychosis or the effectiveness of antipsychotics in relapse prevention.

Using a nationwide Swedish register-linkage approach, integrating several national health and population registers, we examined clinical characteristics, antipsychotic use, and prognosis among individuals with incident amphetamine-related psychosis (ICD-10 F15.5) and FEP with comorbid amphetamine use disorder (F2x+F15). Medication use was modeled into drug use periods utilizing the PRE2DUP method.

Among individuals diagnosed with F15.5, the prevalence of any outpatient antipsychotic use during the first year was 38.4%. Over a mean follow-up of 8.7 years, antipsychotic medication was associated with a 13% reduction in risk for psychosis relapse. Within amphetamine-related groups, the leading causes of death were accidents, suicides, and accidental poisonings.

Amphetamine-related psychosis is associated with poorer mortality prognosis than other psychotic disorders. Antipsychotic use is common post-diagnosis, though its effect on relapse prevention appears modest. Targeted interventions to reduce preventable deaths, such as overdoses and suicides, are needed in this underserved population.

**Disclosure of Interest:**

None Declared

